# Impact of acute kidney injury on mortality in patients with acute variceal bleeding

**DOI:** 10.1186/s12876-021-01862-x

**Published:** 2021-07-13

**Authors:** Jae Heon Kim, Chang Bin Im, Sang Soo Lee, Hankyu Jeon, Jung Woo Choi, Hee Jin Kim, Ra Ri Cha, Hyun Chin Cho, Jae Min Lee, Chang Yoon Ha, Hyun Jin Kim, Tae Hyo Kim, Woon Tae Jung, Ok-Jae Lee

**Affiliations:** 1 Department of Internal Medicine, Gyeongsang National University Hospital, Gyeongsang National University School of Medicine, Jinju, Gyeongnam Republic of Korea; 2 Institute of Health Sciences, Gyeongsang National University, Jinju, Republic of Korea; 3 Department of Internal Medicine, Gyeongsang National University Changwon Hospital, Changwon, Republic of Korea

**Keywords:** Acute kidney injury, Liver cirrhosis, Acute variceal bleeding, Mortality, International Club of Ascites criteria

## Abstract

**Background:**

The effect of acute kidney injury (AKI) on patients with acute variceal bleeding (AVB) using the recently proposed International Club of Ascites (ICA) criteria is unclear. This study aimed to evaluate the incidence of AKI using the ICA criteria and factors associated with the outcomes in cirrhotic patients with AVB.

**Methods:**

This retrospective cohort study included data of cirrhotic patients with AVB from two centers in Korea. The association of the ICA criteria for AKI with 6-week mortality was analyzed through univariate and multivariate analyses using the Cox proportional hazard model.

**Results:**

In total, there were 546 episodes of AVB in 390 patients, of which 425 and 121 episodes were due to esophageal and gastric variceal bleeding, respectively. Moreover, 153 patients fulfilled the ICA criteria for AKI, and 64, 30, 39, and 20 patients were diagnosed with stages 1a, 1b, 2, and 3, respectively. Conversely, 97 patients developed AKI within 42 days as per the conventional criteria. The 6-week mortality rate was significantly higher in patients with ICA-AKI than in patients without ICA-AKI; the occurrence of ICA-AKI was an independent factor for predicting the 6-week mortality.

**Conclusion:**

The ICA criteria could help diagnose renal dysfunction earlier, and presence of AKI is a predictor of mortality in patients with cirrhosis and AVB.

**Supplementary Information:**

The online version contains supplementary material available at 10.1186/s12876-021-01862-x.

## Background

Acute variceal bleeding (AVB) is one of the most life-threatening complications of portal hypertension and a major cause of death in patients with liver cirrhosis [1, 2]. Over the past two decades, therapeutic developments in the management of AVB have resulted in marked improvement in the mortality rate in the initial 6 weeks [3, 4]. However, despite advances in the management approaches for AVB, the 6-week mortality rate remains high at 10–20% [1, 5]. Several factors increase the risk of mortality in patients with AVB: Child–Pugh score, model of end-stage liver disease (MELD) score, hepatocellular carcinoma (HCC), hypovolemic shock, hepatic venous gradient, active bleeding at endoscopy, and renal dysfunction [4, 6–8].

Renal dysfunction occurs in approximately 20% of hospitalized patients with cirrhosis [9–12]. Acute kidney injury (AKI) may have a significant effect on the mortality of patients with liver cirrhosis and AVB. Recently, the International Club of Ascites (ICA) proposed new diagnostic criteria for AKI in patients with cirrhosis [13], which defines AKI as either an increase of baseline serum creatinine level by ≥ 0.3 mg/dL or a 50% increase from baseline levels. A recent study showed that AKI defined using the ICA criteria commonly occurs (41%) in patients with gastric variceal bleeding [14]. In addition, the ICA-AKI criteria could help diagnose renal dysfunction earlier than the conventional criteria and predict mortality in patients with liver cirrhosis and gastric variceal bleeding. This could have a significant effect on mortality in patients with esophageal and gastric variceal bleeding. However, few studies have diagnosed AKI defined according to the ICA criteria in patients with esophageal variceal bleeding. Therefore, this retrospective cohort study aimed to investigate the incidence of AKI as per the new ICA criteria in cirrhotic patients with esophageal and gastric variceal bleeding and to investigate the factors related to the 6-week mortality.

## Material and methods

### Study population

From January 2015 to December 2019, a total of 640 consecutive episodes of AVB in 463 patients with cirrhosis at two centers were screened for enrollment. The inclusion criteria were as follows: (1) aged > 18 years, (2) liver cirrhosis, and (3) acute esophageal or gastric variceal bleeding confirmed by endoscopy. The exclusion criteria were as follows: (1) loss to follow-up within 3 months after variceal bleeding (26 episodes), (2) end-stage renal disease (4 episodes), and (3) recent variceal bleeding within 3 months (64 episodes in 43 patients). Laboratory test results and comorbidities, including diabetes mellitus and hypertension, were retrieved from the medical records of patients with AVB. The patients’ medical histories were reviewed to identify demographic, laboratory, and endoscopic data.

The Institutional Review Boards of Gyeongsang National University Changwon Hospital (IRB No. 2017-07-003) and Gyeongsang National University Hospital (IRB No. 2015-01-008) approved this study. The informed consent was waived due to the retrospective design of this study, as determined by the Institutional Review Boards.

### Definition and treatment

Medical charts were retrospectively reviewed to determine death and AKI occurrence within 6 weeks. For patients with multiple episodes of AVB during the study period, all episodes except AVB episodes within the last 3 months were considered for analyses of 6-week mortality. The index date in this analysis was defined as the date of the first visit to the hospital for AVB. After enrollment, all patients underwent laboratory investigations, including serum creatinine, at least every 2–3 days.

Liver cirrhosis was defined as the (1) presence of clinical signs of portal hypertension manifested as varices, ascites, or hepatic encephalopathy or (2) compatible imaging findings accompanied by thrombocytopenia (< 100,000/µL). Variceal bleeding was defined as hemorrhage from esophageal or gastric varices confirmed by endoscopy. AVB was defined as bleeding in a patient with liver cirrhosis and hematemesis within the last 24 h of presentation and/or ongoing melena within the last 24 h [5]. Active variceal bleeding was defined as spurting or oozing at the time of endoscopy [5].

Endoscopy was performed within 24 h after admission in all patients. Endoscopic variceal ligation (EVL) was performed for treatment of bleeding from esophageal varices or gastroesophageal varices type 1, whereas endoscopic variceal obturation (EVO) with histoacryl was performed for the treatment of bleeding from gastroesophageal varices type 2 or isolated gastric varices. All endoscopic procedures were performed by endoscopists with > 5 years of experience. Balloon-occluded retrograde transvenous obliteration (BRTO) was used in isolated gastric varices as per the clinical decision of the physicians. Prophylactic antibiotics and vasoconstrictors such as terlipressin or somatostatin were started as soon as variceal bleeding occurred. Balloon tamponade was used for refractory esophageal bleeding despite immediate endoscopic therapy. However, emergency intrahepatic portosystemic shunt was not available in our centers.

Failure to control bleeding was defined as death or the need to change treatment defined by one of the following events: (1) fresh hematemesis or nasogastric aspiration of ≥ 100 mL of fresh blood ≥ 2 h after the start of a therapeutic endoscopy, (2) development of hypovolemic shock, or (3) 3 g drop in hemoglobin within 24 h if no transfusion is administered [1, 15]. Variceal rebleeding was defined as recurrent melena or hematemesis after day 5 in any of the following criteria: (1) hospital admission, (2) blood transfusion, (3) 3 g drop in hemoglobin, or (4) death within 6 weeks [15, 16].

### Acute kidney injury

The ICA criteria defined AKI as an increase of ≥ 0.3 mg/dL within 48 h or ≥ 50% of baseline in serum creatinine levels within the previous 7 days [13]. Baseline serum creatinine was defined as the most recent value of serum creatinine within 3 months before admission, because serum creatinine before admission was barely available in patients developing AVB. In patients without serum creatinine value available in the 3 months before admission, the last stable level of serum creatinine between 3 months and 1 year was used as the baseline. If preadmission serum creatinine values were not available, the creatinine level at admission was used as the baseline [9, 11]. Conventional AKI was defined as a 50% increase in serum creatinine with a final value above 1.5 mg/dL.

AKI stages were defined as follows [13]: AKI stage 1, an increase in serum creatinine ≥ 0.3 mg/dL or increase in serum creatinine ≥ 1.5-fold to twofold from baseline; AKI stage 2, an increase in serum creatinine by > 2 to threefold from baseline; and AKI stage 3, an increase in serum creatinine by > threefold from baseline or serum creatinine ≥ 4.0 mg/dL with an acute increase of 0.3 mg/dL or need for renal replacement therapy (RRT). Patients in stage 1 were further subdivided into stage 1a (serum creatinine < 1.5 mg/dL) and stage 1b (serum creatinine ≥ 1.5 mg/dL) [12]. If the patients experienced multiple episodes of AKI within 42 days, only the first episode was considered. AKI progression was defined as progression to a higher AKI stage and/or need for RRT during hospitalization.

The cause of AKI was classified into three groups: (1) pre-renal AKI, (2) hepatorenal syndrome (HRS)-AKI, and (3) intrinsic-renal AKI. The diagnostic criteria of HRS were defined using the ICA criteria [13].

### Statistical analysis

The Mann–Whitney U test was used to analyze differences in continuous variables between groups. Fisher’s exact and Pearson’s chi-square tests were used to analyze qualitative data. Survival curves for the development of 6-week survival were calculated using the Kaplan–Meier method and compared using the log-rank test. Univariate and multivariate analyses were conducted using the Cox proportional regression model to identify potential factors associated with AKI development and 6-week mortality. The components of the Child–Pugh scores and MELD scores were excluded in the multivariate analyses to avoid collinearity. The risk was expressed as hazard ratio (HR) and 95% confidence interval (CI). A two-sided *P* value of < 0.05 was considered significant for all analyses. Statistical analyses were conducted using the PASW software (version 18; SPSS Inc, Chicago, IL).

## Results

### Patient characteristics

**Fig. 1:**
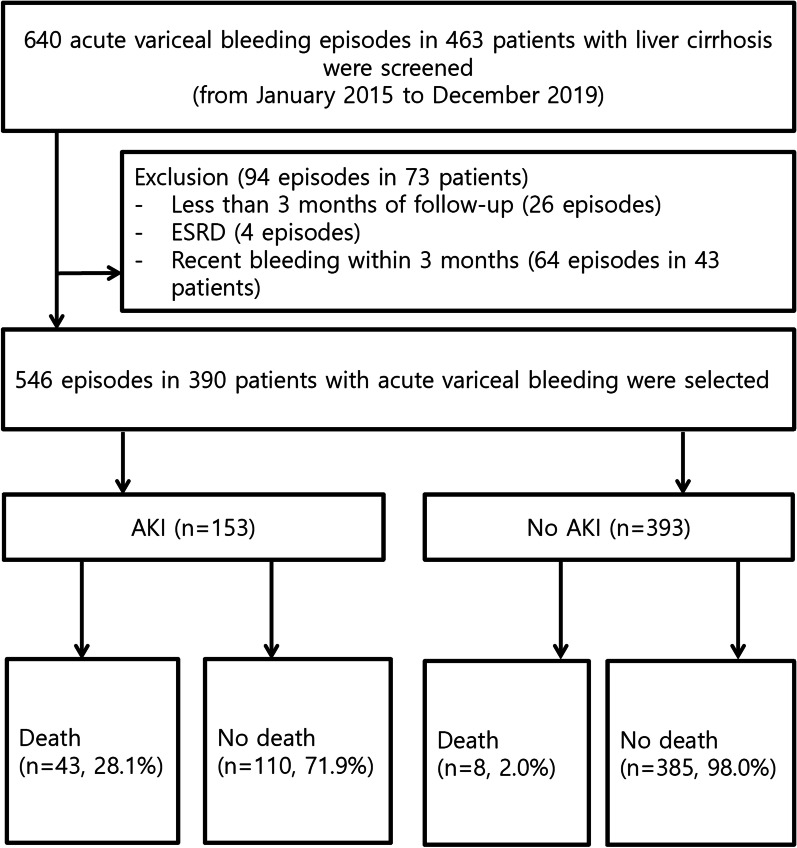
Flow chart. AKI, acute kidney injury; ESRD, end-stage renal disease

In total, 546 episodes in 390 patients with AVB were selected for analysis (Fig. 1). Baseline characteristics of 546 episodes with AVB are shown in Table 1. The median patient age was 58 years, and 84.1% of the patients were male. The primary etiologies of cirrhosis were alcohol consumption (64.7%), hepatitis B virus (19.8%), hepatitis C virus (7.1%), and cryptogenic causes (8.4%). Bleeding sources were esophageal varices in 425 (77.8%) and gastric varices in 121 (22.2%) patients. EVL, EVO, and BRTO were performed in 450, 90, and 6 patients with AVB, respectively. Initial endoscopy revealed active variceal bleeding in 232 patients (42.5%). Failure to control bleeding was noted in 32 patients (5.9%). Variceal rebleeding was observed in 38 patients (7.0%) after day 5.

**Table 1:** Baseline characteristics of patients with acute variceal bleeding (n = 546)

	All patients (n = 546)	No AKI (n = 393)	AKI (n = 153)	*P*
Age, year	58.0 (51.0–68.0)	58.0 (51.5–68.0)	58.0 (51.0–67.0)	0.810
Male sex	459 (84.1%)	326 (83.0%)	133 (86.9%)	0.298
Bleeding source				0.360
Esophageal varices	425 (77.8%)	310 (78.9%)	115 (75.2%)	
Gastric varices	121 (22.2%)	83 (21.1%)	38 (24.8%)	
Active bleeding at endoscopy	232 (42.5%)	161 (41.0%)	71 (46.4%)	0.250
Etiology				0.538
Alcohol	353 (64.7%)	247 (62.8%)	106 (69.3%)	
HBV	108 (19.8%)	83 (21.1%)	25 (16.3%)	
HCV	39 (7.1%)	29 (7.4%)	10 (6.5%)	
Cryptogenic	46 (8.4%)	34 (8.7%)	12 (7.8%)	
BMI, kg/m^2^	22.9 (20.7–25.0)	22.9 (20.8–24.9)	23.1 (20.5–25.3)	0.602
Diabetes	184 (33.7%)	128 (32.6%)	56 (36.6%)	0.420
HCC	82 (15.0%)	44 (11.2%)	38 (24.8%)	< 0.001
Child–Pugh classification				< 0.001
A	158 (28.9%)	133 (33.8%)	25 (16.3%)	
B	265 (48.5%)	198 (50.4%)	67 (43.8%)	
C	123 (22.5%)	62 (15.8%)	61 (39.9%)	
MELD score	12.0 (10.0–18.0)	11.0 (9.0–14.0)	19.0 (14.0–25.0)	< 0.001
Ascites	293 (53.7%)	199 (50.6%)	94 (61.4%)	0.028
Encephalopathy	49 (9.0%)	23 (5.9%)	26 (17.0%)	< 0.001
Diuretics	177 (32.5%)	116 (29.6%)	61 (39.9%)	0.025
Hemoglobin, g/dL	8.7 (7.5–10.0)	9.0 (7.7–10.2)	8.0 (6.9–9.0)	< 0.001
Platelet, × 10^9^/L	84.5 (59.0–118.3)	84.0 (69.5–113.0)	85.0 (55.0–133.0)	0.602
PT-INR	1.43 (1.26–1.69)	1.37 (1.25–1.57)	1.69 (1.42–2.08)	< 0.001
Bilirubin, mg/dL	1.43 (0.89–2.67)	1.30 (0.83–2.30)	1.87 (1.01–4.00)	< 0.001
Albumin, g/dL	2.9 (2.5–3.4)	3.0 (2.6–3.4)	2.7 (2.2–3.0)	< 0.001
sCr at admission, mg/dL	0.79 (0.64–1.00)	0.73 (0.63–0.89)	1.12 (0.84–1.58)	< 0.001
Baseline sCr, mg/dL	0.71 (0.61–0.86)	0.71 (0.60–0.84)	0.75 (0.62–0.92)	0.009
Failure to control bleeding	32 (5.9%)	11 (2.8%)	21 (13.7%)	< 0.001
Variceal rebleeding	38 (7.0%)	27 (6.9%)	11 (7.2%)	0.854

HBV, hepatitis B virus; HCV, hepatitis C virus; BMI, body mass index; HCC, hepatocellular carcinoma; MELD score, Model For End-Stage Liver Disease score; PT-INR, prothrombin time-international normalized ratio. Data are presented as median (interquartile range) for continuous data and percentages for categorical data

### AKI development

**Fig. 2:**
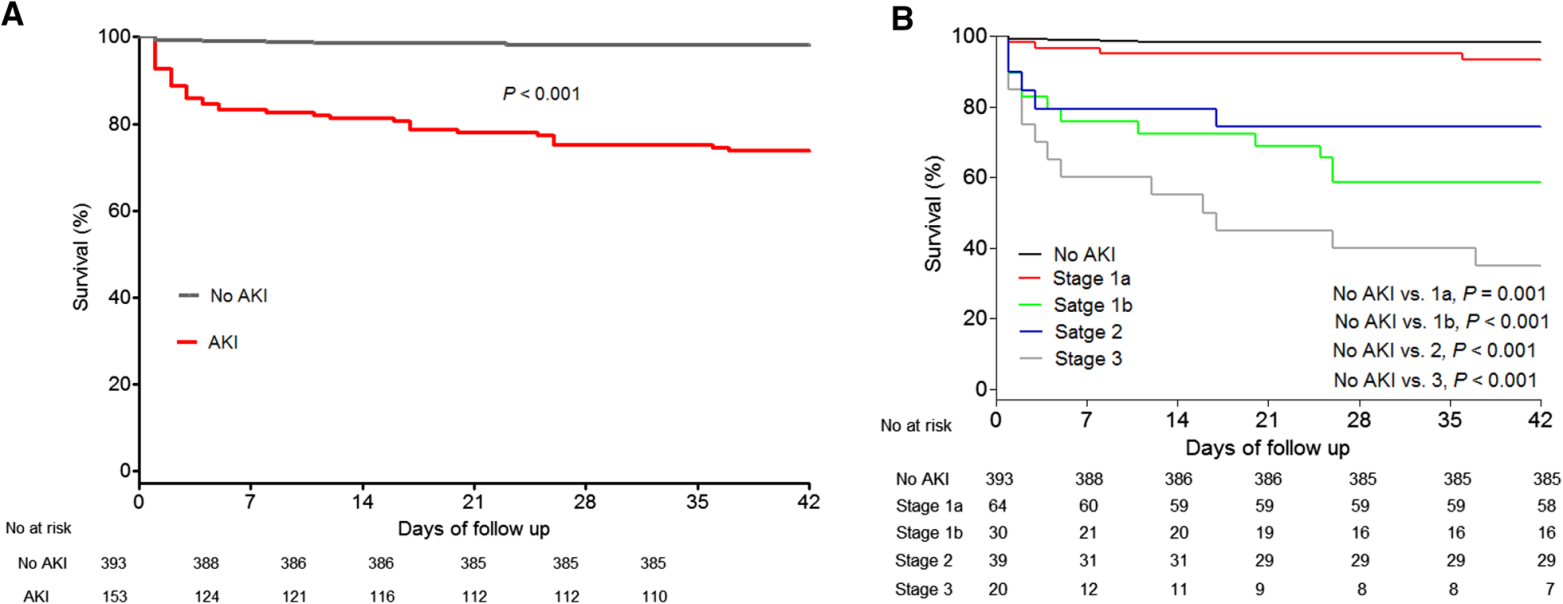
Six-week mortality in patients diagnosed with acute kidney injury (AKI) using the International Club of Ascites (ICA) criteria (n = 546). **A** Patients without AKI show better survival than those with AKI (*P* < 0.001). **B** Patients with AKI stages 1a (*P* = 0.001), 1b (*P* < 0.001), 2 (*P* < 0.001), and 3 (*P* < 0.001) had higher 6-week mortality rate at the time of AKI diagnosis than patients without AKI. AKI, acute kidney injury; ICA, International Club of Ascites

Of the 546 episodes, 153 (28.0%) fulfilled the ICA criteria for AKI. The composition of baseline creatinine is shown in Additional file 1: Table S1. AKI was present in 128 (83.7%) patients at admission, whereas 7 (4.6%) had AKI beyond 5 days of hospitalization. Patients with AKI had higher median serum creatinine level at admission (1.12 mg/dL vs. 0.73 mg/dL; *P* < 0.001) and baseline serum creatinine levels (0.75 mg/dL vs. 0.71 mg/dL; *P* < 0.009) than patients without AKI. At the time of AKI diagnosis, 64 (41.8%), 30 (19.6%), 39 (25.5%), and 20 (13.1%) patients had initial AKI at stages 1a, 1b, 2, and 3, respectively (Table 2). The median serum creatinine levels at the time of AKI diagnosis were 1.19 mg/dL, 2.03 mg/dL, 1.70 mg/dL, and 2.70 ng/dL in stages 1a, 1b, 2, and 3, respectively. During hospitalization, peak AKI stages 1a, 1b, 2, and 3 were seen in 56 (36.6%), 27 (17.6%), 37 (24.2%), and 33 (21.6%) patients, respectively. Among the 153 patients with AKI, AKI progression was observed in 30 (19.6%) patients, and 19 (12.4%) patients needed RRT. The 6-week mortalities as per the progression of AKI are shown in Fig. 2. In 153 patients with AKI, progression of AKI was markedly more common among non-survivors (53.5%) than among survivors (6.4%; *P* < 0.001) (Table 2). The causes of AKI were (1) prerenal AKI in 131 (85.6%) patients, (2) HRS-AKI in 18 (11.8%) patients, and (3) intrinsic renal AKI in 4 (2.6%) patients (Table 2).

**Table 2:** Renal factors of six-week mortality in patients with AKI (n = 153)

	All patients (n = 153)	No Death (n = 110)	Death (n = 43)	*P*
Baseline sCr, mg/dL	0.75 (0.62–0.93)	0.71 (0.59–0.83)	0.91 (0.72–1.19)	< 0.001
sCr at admission, mg/dL	1.12 (0.84–1.58)	1.13 (0.83–1.55)	1.05 (0.87–1.65)	0.761
sCr at initial AKI	1.46 (1.18–2.03)	1.32 (1.13–1.74)	2.05 (1.50–2.80)	< 0.001
Peak sCr, mg/dL	1.58 (1.20–2.32)	1.36 (1.14–1.77)	2.67 (1.91–3.76)	< 0.001
Initial AKI stage				< 0.001
Stage 1a	64 (41.8%)	58 (52.7%)	6 (14.0%)	
Stage 1b	30 (19.6%)	16 (14.5%)	14 (32.6%)	
Stage 2	39 (25.5%)	29 (26.4%)	10 (23.3%)	
Stage 3	20 (13.1%)	7 (6.4%)	13 (30.2%)	
Progression of AKI	30 (19.6%)	7 (6.4%)	23 (53.5%)	< 0.001
Need for RRT	19 (12.4%)	1 (0.9%)	18 (41.9%)	< 0.001
Peak AKI stage				< 0.001
Stage 1a	56 (36.6%)	53 (48.2%)	3 (7.0%)	
Stage 1b	27 (17.6%)	19 (17.3%)	8 (18.6%)	
Stage 2	37 (24.2%)	30 (27.3%)	7 (16.3%)	
Stage 3	33 (21.6%)	8 (7.3%)	25 (58.1%)	
Cause of AKI				< 0.001
Prerenal AKI	131 (85.6%)	104 (94.5%)	27 (62.8%)	
HRS	18 (11.8%)	3 (2.7%)	15 (34.9%)	
Intrinsic	4 (2.6%)	3 (2.7%)	1 (2.3%)	

sCr, serum creatinine; AKI, acute kidney injury, RRT; renal replace therapy, HRS, hepatorenal syndrome

Data are presented as median (interquartile range) for continuous data and percentages for categorical data

In the univariate analysis, risk factors associated with AKI development were HCC (HR, 1.95; *P* < 0.001), failure to control bleeding (HR, 2.78; *P* < 0.001), use of diuretics (HR, 1.39; *P* = 0.048), initial hemoglobin level (HR, 0.83 per g/dL; *P* < 0.001), Child–Pugh score (HR, 1.26 per point; *P* < 0.001), and MELD score (HR, 1.10 per point; *P* < 0.001). In the multivariate analysis for AKI development, the risk factors were HCC (HR, 1.70; *P* = 0.007), hemoglobin level (HR, 0.91 per g/dL; *P* = 0.026), and MELD score (HR, 1.11 per point; *P* < 0.001) (Table 3).

**Table 3:** Univariate and multivariate analyses showing significant predictive factors of acute kidney injury (n = 546)

Variable	Univariate analysis		Multivariate analysis	
	*P*	HR (95% CI)	*P*	HR (95% CI)
Male	0.318	1.27 (0.79–2.03)		
Age per year	0.950	1.00 (0.99–1.01)		
HCC	< 0.001	1.95 (1.35–2.82)	**0.007**	1.70 (1.16–2.49)
Failure to control bleeding	< 0.001	2.78 (1.75–4.41)	0.202	1.37 (0.84–2.23)
Diuretics	0.048	1.39 (1.00–1.92)	0.398	1.18 (0.81–1.72)
Initial hemoglobin per g/dL	< 0.001	0.83 (0.77–0.91)	**0.026**	0.91 (0.83–0.99)
Child–Pugh score per point	< 0.001	1.26 (1.18–1.36)	0.115	0.91 (0.81–1.02)
MELD score per point	< 0.001	1.10 (1.08–1.12)	** < 0.001**	1.11 (1.08–1.14)

Bold numbers indicate significant P-values in multivariate analysis

HR, hazard ratio; CI, confidence interval; HCC, hepatocellular carcinoma; MELD score, model for end-stage liver disease score

### Effect of AKI on survival

**Fig. 3:**
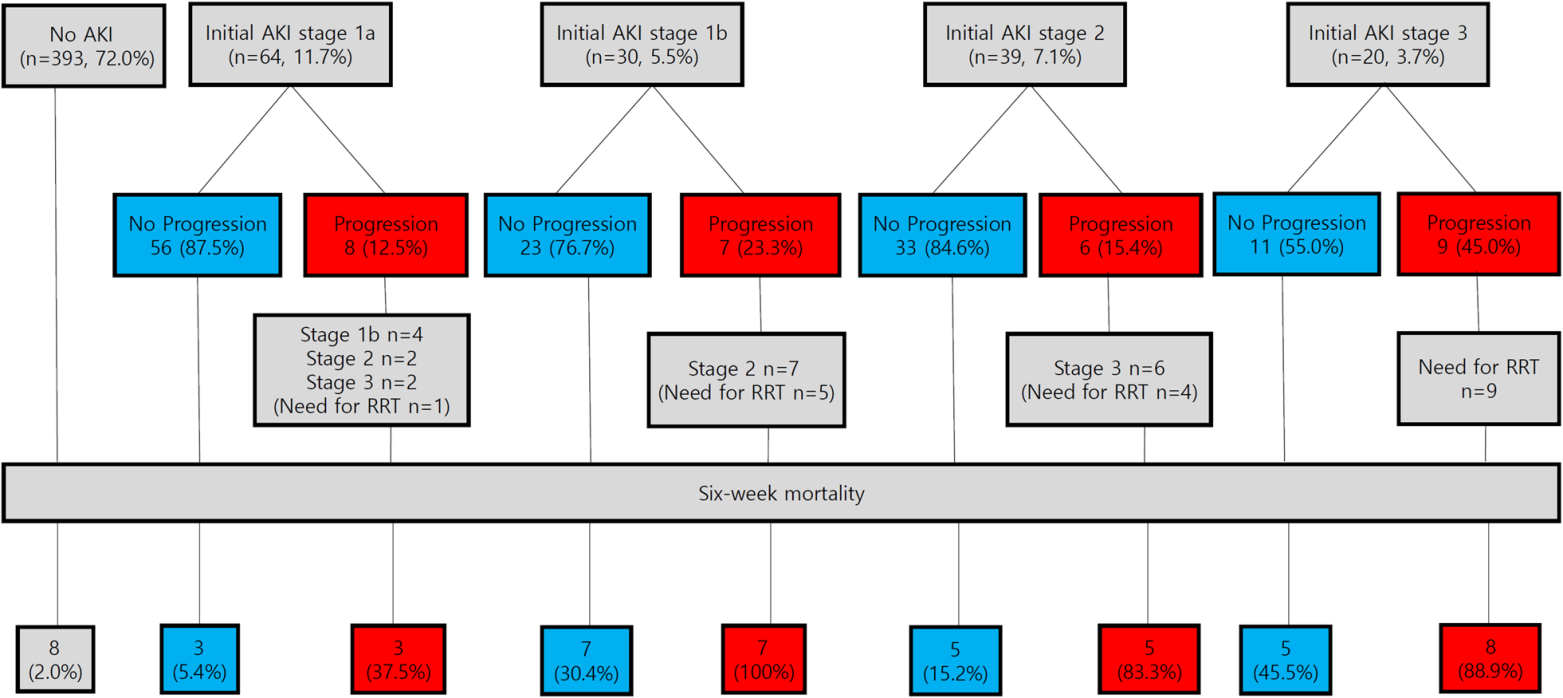
Course of acute kidney injury (n = 546). At the time of acute kidney injury (AKI) diagnosis, patients with higher AKI stages have a higher 6-week mortality rate than those with lower stages. Patients with AKI progression had higher 6-week mortality rate than those without AKI progression

During the study period, the overall mortality rate was 9.3% (n = 51) at 6 weeks. The 6-week mortality rate was higher in patients with AKI (28.1%) than in patients without AKI (2.0%, *P* < 0.001) (Fig. 2A). In addition, patients with initial AKI stage 1a (9.4%; *P* = 0.001), stage 1b (46.7%; *P* < 0.001), stage 2 (25.6%; *P* < 0.001), and stage 3 (65.0%; *P* < 0.001) had higher 6-week mortality rates (Fig. 2B) than those without AKI (2.0%). Regardless of the initial AKI stage, patients with AKI progression had higher 6-week mortality rate than those without AKI progression (Fig. 3). Patients with AKI stage 1b did not have a significantly higher 6-week mortality rate (46.7%; *P* = 0.080) than those with initial AKI stage 2 (25.6%). Moreover, the median serum creatinine level at diagnosis in patients with initial AKI 1b (2.03 mg/dL) was higher than that in those with stage 2 (1.70 mg/dL; *P* < 0.001). According to the conventional diagnostic criteria of AKI, 97 (17.8%) patients developed AKI within 42 days. Patients with conventional AKI diagnosis had higher 6-week mortality rate (n = 40, 41.2%; *P* < 0.001) than those without (n = 11, 2.4%). There were 56 patients who did not meet the conventional AKI criteria but did meet the new ICA-AKI criteria. All 56 of these patients had AKI stage 1b, with 3 of the 56 dying within 42 days.

Factors associated with 6-week mortality on the univariate analysis were HCC (HR, 4.33; *P* < 0.001), failure to control bleeding (HR, 14.03; *P* < 0.001), initial hemoglobin level (HR, 0.85 per g/dL; *P* = 0.031), Child–Pugh score (HR, 1.62 per point; *P* < 0.001), MELD score (HR, 1.17 per point; *P* < 0.001), and AKI (HR 15.69; *P* < 0.001). Factors associated with 6-week mortality in the multivariate analysis were HCC (HR, 3.29; *P* < 0.001), failure to control bleeding (HR, 4.95; *P* < 0.001), MELD score (HR, 1.10 per point; *P* < 0.001), and AKI (HR, 3.95; *P* = 0.002) (Table 4) (Additional file 1: Table S2).

**Table 4:** Univariate and multivariate analyses showing significant predictive factors of six-week mortality (n = 546)

Variable	Univariate analysis		Multivariate analysis	
	*P*	HR (95% CI)	*P*	HR (95% CI)
Male	0.627	1.22 (0.55–2.71)		
Age per year	0.594	1.01 (0.98–1.03)		
HCC	< 0.001	4.33 (2.48–7.56)	** < 0.001**	3.29 (1.83–5.94)
Failure to control bleeding	< 0.001	14.03 (7.92–24.92)	** < 0.001**	4.95 (2.69–9.09)
Initial hemoglobin per g/dL	0.031	0.85 (0.73–0.98)	0.400	1.07 (0.92–1.25)
Child–Pugh score per point	< 0.001	1.62 (1.43–1.84)	0.066	1.17 (0.99–1.39)
MELD score per point	< 0.001	1.17 (1.13–1.20)	** < 0.001**	1.10 (1.05–1.15)
AKI	< 0.001	15.69 (7.37–33.39)	**0.002**	3.95 (1.65–9.51)

Bold numbers indicate significant P-values in multivariate analysis

HR, hazard ratio; CI, confidence interval; HCC, hepatocellular carcinoma; MELD score, model for end-stage liver disease score; AKI, acute kidney injury

## Discussion

In this study, the incidence of AKI according to the new ICA criteria was 28% in 546 patients with AVB, whereas the incidence according to the conventional criteria was 17.8%. This study showed that HCC, initial hemoglobin level, and MELD score are risk factors for development of ICA-AKI. Further, the occurrence of ICA-AKI is an independent risk factor for 6-week mortality. In particular, among patients with AKI, those with a higher initial AKI stage and with AKI progression showed higher mortality rate than those with a lower initial AKI stage and without AKI progression.

Recent studies have reported renal impairment in 19%-41% of patients with cirrhosis and AVB [14, 17–20]. Of these studies, only two small-scale have studies used the new AKI diagnostic criteria [14, 20]. In the most recent study in China, large-scale retrospective results using ICA criteria were published [21]. However, the incidence rates of ICA-AKI and conventional AKI in cirrhotic patients with acute gastrointestinal bleeding were 7.1% and 5.0%, respectively, which are very low compared with those in previous studies. In addition, the mortality rate was significantly increased by renal dysfunction and ICA-AKI stages 1B, 2, and 3 (14.5% vs. 2.2%, *P* < 0.001), but not by any stage ICA-AKI (11.1% vs. 2.8%, *P* = 0.083). This study, which included 546 episodes in patients diagnosed with cirrhosis and active gastroesophageal variceal bleeding, showed an overall incidence of 28% and 17.8% for AKI diagnosed using the ICA and conventional criteria within 42 days, respectively. A previous meta-analysis focusing on gastrointestinal bleeding in patients with cirrhosis reported an incidence of 21% of renal dysfunction, but the incidence of renal dysfunction according to the new AKI diagnostic criteria was up to 25% [22]. These findings suggest that the ICA-AKI criteria can detect renal dysfunction in patients with AVB earlier than the conventional criteria. However, in our study, 3 of the 56 patients (serum creatinine < 1.5 mg/dL) diagnosed with ICA-AKI and who did not meet the conventional criteria died.

We identified that AKI development in patients with cirrhosis and AVB was associated with a significant increase in the 6-week mortality rate. The 6-week mortality rate of patients with AKI was 28.1%. In addition, there was a 3.0-fold increase in the risk of 6-week mortality rate in patients with AKI than in those without AKI. A previous meta-analysis suggested that renal dysfunction increased the risk of mortality among patients with cirrhosis and acute gastrointestinal bleeding [22]. In a previous study of 113 patients with cirrhosis and gastric variceal bleeding conducted by Hsieh [14], the 6-week mortality rate in patients with ICA-AKI (37%) was higher than that in those without ICA-AKI (3%, *P* < 0.001), and AKI stages were independent predictors of 3-month survival. In another study of 132 patients with acute gastric variceal bleeding, the 6-week mortality rate was higher with conventional AKI criteria (63.6%) than that without conventional AKI (7.3%), and AKI defined using conventional criteria was an independent predictive factor for 6-week mortality. However, few recent studies have demonstrated a relationship between AKI and mortality in patients with cirrhosis and acute gastroesophageal variceal bleeding. To the best of our knowledge, this is one of the largest studies that have demonstrated the incidence of ICA-AKI and the relationship between ICA-AKI and 6-week mortality in patients with acute gastroesophageal variceal bleeding.

The mechanism of renal impairment in cirrhosis is primarily related to the development of circulatory dysfunction [23, 24]. Systemic vascular resistance is reduced by primary arterial vasodilatation in the splanchnic circulation following activation of the vasoconstrictor system including the sympathetic nervous system, renin–angiotensin–aldosterone system, and arginine-vasopressin secretion. This explains some of the cardinal mechanisms of the development of risk factors for AKI, such as renal retention of sodium, ascites formation, and HRS. Hypovolemia as a consequence of AVB is a common cause of impaired renal function in cirrhosis. In our study, 53.7% of the total patients had ascites at the time of bleeding. The independent predictors for AKI development were HCC, initial hemoglobin level, and MELD score.

This study was limited by its retrospective nature, and we were unable to regularly collate data on factors related to AKI and mortality in all patients. Further, our results did not show whether early detection of renal dysfunction using the ICA-AKI criteria could result in a better prognosis in patients with cirrhosis and AVB. We only found that the 6-week mortality rate in patients with initial AKI stage 1a (serum creatinine < 1.5 mg/dL) was higher than that in patients without ICA-AKI. In addition, we did not prospectively perform volume expansion for pre-renal AKI or HRS-specific treatment when patients were diagnosed early with AKI stage 1a. Therefore, it was not possible to demonstrate whether early treatment of AKI affected survival. Despite these limitations, this study was conducted with similar treatment protocols by physicians from two institutions affiliated with the same university, and we had sufficient creatinine data for almost all patients. Certainly, high-quality prospective studies are needed to validate the role of ICA-AKI in prognosis in patients with AVB.

## Conclusion

AKI events defined by the ICA criteria are common in patients with cirrhosis and AVB, and the occurrence of AKI is an important predictor of 6-week mortality. Our findings suggest that the ICA-AKI criteria can detect renal dysfunction earlier in cirrhotic patients with AVB, but the role of early detection of ICA-AKI in prognosis warrants further investigations in patients with AVB.

## Supplementary Information

**Additional file 1**. **Supp. Table 1.** Composition of baseline creatinine in 153 patients with ICA-AKI. **Supp. Table 2.** Multivariate analyses showing significant predictive factors of 42-day mortality (n = 546).

## Data Availability

The datasets generated and/or analyzed during the present study are not publicly available due to ethical and confidentiality reasons but are available from the corresponding author on reasonable request under the Gyeongsang National University Hospital Ethics Committee’s approval. The data that support the findings of this study are available on request to the corresponding author (Sang Soo Lee, Email: 3939lee@naver.com).

## Abbreviations

AVB: Acute variceal bleeding
MELD: Model for end-stage liver disease
HCC: Hepatocellular carcinoma
AKI: Acute kidney injury
ICA: International Club of Ascites
EVL: Endoscopic variceal ligation
EVO: Endoscopic variceal obturation
BRTO: Balloon-occluded retrograde transvenous obliteration
HRS: Hepatorenal syndrome
CI: Confidence interval
HR: Hazard ratio

## References

[1] de Franchis R, Baveno VIF (2015). Expanding consensus in portal hypertension: report of the Baveno VI Consensus Workshop: Stratifying risk and individualizing care for portal hypertension. J Hepatol.

[2] Graham DY, Smith JL (1981). The course of patients after variceal hemorrhage. Gastroenterology.

[3] Chalasani N, Kahi C, Francois F, Pinto A, Marathe A, Bini EJ, Pandya P, Sitaraman S, Shen J (2003). Improved patient survival after acute variceal bleeding: a multicenter, cohort study. Am J Gastroenterol.

[4] Augustin S, Muntaner L, Altamirano JT, Gonzalez A, Saperas E, Dot J, Abu-Suboh M, Armengol JR, Malagelada JR, Esteban R (2009). Predicting early mortality after acute variceal hemorrhage based on classification and regression tree analysis. Clin Gastroenterol Hepatol.

[5] Sarin SK, Kumar A, Angus PW, Baijal SS, Baik SK, Bayraktar Y, Chawla YK, Choudhuri G, Chung JW, de Franchis R (2011). Diagnosis and management of acute variceal bleeding: Asian Pacific Association for Study of the Liver recommendations. Hepatol Int.

[6] Bambha K, Kim WR, Pedersen R, Bida JP, Kremers WK, Kamath PS (2008). Predictors of early re-bleeding and mortality after acute variceal haemorrhage in patients with cirrhosis. Gut.

[7] Abraldes JG, Villanueva C, Banares R, Aracil C, Catalina MV, Garci APJC, Bosch J, Spanish Cooperative Group for Portal H, Variceal B (2008). Hepatic venous pressure gradient and prognosis in patients with acute variceal bleeding treated with pharmacologic and endoscopic therapy. J Hepatol.

[8] Amitrano L, Guardascione MA, Bennato R, Manguso F, Balzano A (2005). MELD score and hepatocellular carcinoma identify patients at different risk of short-term mortality among cirrhotics bleeding from esophageal varices. J Hepatol.

[9] Fagundes C, Barreto R, Guevara M, Garcia E, Sola E, Rodriguez E, Graupera I, Ariza X, Pereira G, Alfaro I (2013). A modified acute kidney injury classification for diagnosis and risk stratification of impairment of kidney function in cirrhosis. J Hepatol.

[10] Piano S, Rosi S, Maresio G, Fasolato S, Cavallin M, Romano A, Morando F, Gola E, Frigo AC, Gatta A (2013). Evaluation of the Acute Kidney Injury Network criteria in hospitalized patients with cirrhosis and ascites. J Hepatol.

[11] Belcher JM, Garcia-Tsao G, Sanyal AJ, Bhogal H, Lim JK, Ansari N, Coca SG, Parikh CR, Consortium T-A (2013). Association of AKI with mortality and complications in hospitalized patients with cirrhosis. Hepatology.

[12] Huelin P, Piano S, Sola E, Stanco M, Sole C, Moreira R, Pose E, Fasolato S, Fabrellas N, de Prada G (2017). Validation of a staging system for acute kidney injury in patients with cirrhosis and association with acute-on-chronic liver failure. Clin Gastroenterol Hepatol.

[13] Angeli P, Gines P, Wong F, Bernardi M, Boyer TD, Gerbes A, Moreau R, Jalan R, Sarin SK, Piano S (2015). Diagnosis and management of acute kidney injury in patients with cirrhosis: revised consensus recommendations of the International Club of Ascites. J Hepatol.

[14] Hsieh YC, Lee KC, Chen PH, Su CW, Hou MC, Lin HC (2017). Acute kidney injury predicts mortality in cirrhotic patients with gastric variceal bleeding. J Gastroenterol Hepatol.

[15] Tripathi D, Stanley AJ, Hayes PC, Patch D, Millson C, Mehrzad H, Austin A, Ferguson JW, Olliff SP, Hudson M (2015). U.K. guidelines on the management of variceal haemorrhage in cirrhotic patients. Gut.

[16] de Franchis R, Baveno VF (2010). Revising consensus in portal hypertension: report of the Baveno V consensus workshop on methodology of diagnosis and therapy in portal hypertension. J Hepatol.

[17] Fallatah HI, Al Nahdi H, Al Khatabi M, Akbar HO, Qari YA, Sibiani AR, Bazaraa S (2012). Variceal hemorrhage: Saudi tertiary center experience of clinical presentations, complications and mortality. World J Hepatol.

[18] Teng W, Chen WT, Ho YP, Jeng WJ, Huang CH, Chen YC, Lin SM, Chiu CT, Lin CY, Sheen IS (2014). Predictors of mortality within 6 weeks after treatment of gastric variceal bleeding in cirrhotic patients. Medicine (Baltimore).

[19] Hung TH, Tsai CC, Tsen KG, Hsieh YH, Tseng CW (2016). No mortality difference following treatment with terlipressin or somatostatin in cirrhotic patients with gastric variceal hemorrhage. Saudi J Gastroenterol.

[20] Vilela EG, Pinheiro CDS, Saturnino SF, Gomes CGO, Nascimento VCD, Andrade MVM (2018). Evaluation of the behavior of levels of Hmgb1 and Il6 as predictors of infection, acute kidney injury and mortality in cirrhotic patients with variceal bleeding. Arq Gastroenterol.

[21] Xu X, Liu B, Lin S, Li B, Wu Y, Li Y, Zhu Q, Yang Y, Tang S, Meng F (2020). Terlipressin may decrease in-hospital mortality of cirrhotic patients with acute gastrointestinal bleeding and renal dysfunction: a retrospective multicenter observational study. Adv Ther.

[22] Bai Z, Primignani M, Guo X, Zheng K, Li H, Qi X (2019). Incidence and mortality of renal dysfunction in cirrhotic patients with acute gastrointestinal bleeding: a systematic review and meta-analysis. Expert Rev Gastroenterol Hepatol.

[23] Gines P, Schrier RW (2009). Renal failure in cirrhosis. N Engl J Med.

[24] European Association for the Study of the Liver, Electronic address eee, European Association for the Study of the L (2018). EASL Clinical Practice Guidelines for the management of patients with decompensated cirrhosis. J Hepatol.

