# Supplementary material for: Impact of acute kidney injury on mortality in patients with acute variceal bleeding
Source: BMC Gastroenterol. 2021 Jul 13;21:290. doi: 10.1186/s12876-021-01862-x (PMC8276463; doi:10.1186/s12876-021-01862-x)
Supplement: Supplementary file 1 — Additional file 1. Supp. Table 1. Composition of baseline creatinine in 153 patients with ICA-AKI. Supp. Table 2. Multivariate analyses showing significant predictive factors of 42-day mortality (n = 546). [file 12876_2021_1862_MOESM1_ESM.docx]

Supp. Table 1. Composition of baseline creatinine in 153 patients with ICA-AKI

|  | N | % |
| --- | --- | --- |
| 0.3 mg/dl within 48 h | 74 | 48.4 |
| increase of creatinine >50% from baseline within 3 mo | 51 | 33.3 |
| increase of creatinine >50% between 3 mo and 12 mo | 11 | 7.2 |
| No serum creatinine value available within the previous year | 17 | 11.1 |

Supp. Table 2. Multivariate analyses showing significant predictive factors of 42-day mortality (n=546)

| Variable | Univariate analysis | | Multivariate analysis,  model 1 | | Multivariate analysis,  model 2 | | Multivariate analysis,  model 3 | |
| --- | --- | --- | --- | --- | --- | --- | --- | --- |
|  | *P* | HR (95% CI) | *P* | HR (95% CI) | *P* | HR (95% CI) | *P* | HR (95% CI) |
| HCC | <0.001 | 4.33 (2.48-7.56) | **<0.001** | 3.29 (1.83-5.94) | **<0.001** | 2.99 (1.64 - 5.47) | **<0.001** | 4.17 (2.20 - 7.89) |
| Failure to control bleeding | <0.001 | 14.03 (7.92-24.92) | **<0.001** | 4.95 (2.69-9.09) | **<0.001** | 4.60 (2.50 - 8.49) | **<0.001** | 5.74 (3.01 - 10.94) |
| Initial hemoglobin per *g/dL* | 0.031 | 0.85 (0.73-0.98) | 0.400 | 1.07 (0.92 - 1.25) | 0.490 | 1.06 (0.91 - 1.23) | 0.693 | 1.03 (0.89 - 1.20) |
| Child-Pugh score per point | <0.001 | 1.62 (1.43-1.84) | 0.066 | 1.17 (0.99-1.39) | 0.072 | 1.17 (0.99 - 1.40) | 0.117 | 1.17 (0.96 - 1.41) |
| MELD score per point | <0.001 | 1.17 (1.13-1.20) | **<0.001** | 1.10 (1.05-1.15) | **<0.001** | 1.09 (1.04 - 1.14) | **<0.001** | 1.14 (1.09 - 1.19) |
| ICA-AKI | <0.001 | 15.69 (7.37-33.39) | **0.002** | 3.95 (1.65-9.51) |  |  |  |  |
| Conventional AKI | <0.001 | 20.32 (10.12 – 39.67) |  |  | **0.001** | 4.03 (1.71 - 9.52) |  |  |
| Baseline creatinine | <0.001 | 5.52 (3.54 – 8.61) |  |  |  |  | 0.958 | 0.98 (0.53 - 1.18) |

Abbreviations: HR, hazard ratio; CI, confidence interval; HCC, hepatocellular carcinoma; MELD score, model for end-stage liver disease score; AKI, acute kidney injury.

Multivariate analysis model 1 includes ICA-AKI, whereas model 2 and model 3 include conventional AKI and baseline creatinine, respectively.
